# Modulation of Cytochrome P450 2A5 Activity by Lipopolysaccharide: Low-Dose Effects and Non-Monotonic Dose-Response Relationship

**DOI:** 10.1371/journal.pone.0117842

**Published:** 2015-01-30

**Authors:** Ana C. A. X. De-Oliveira, Kátia S. Poça, Paulo R. R. Totino, Francisco J. R. Paumgartten

**Affiliations:** 1 Laboratory of Environmental Toxicology, Department of Biological Sciences, National School of Public Health, Oswaldo Cruz Institute, Oswaldo Cruz Foundation, Rio de Janeiro, RJ, 21040–361, Brazil; 2 Laboratory of Malaria Research, Oswaldo Cruz Institute, Oswaldo Cruz Foundation, Rio de Janeiro, RJ, 21040–361, Brazil; French National Centre for Scientific Research, FRANCE

## Abstract

Mouse cytochrome P450 (CYP) 2A5 is induced by inflammatory conditions and infectious diseases that down-regulate the expression and activity of most other CYP isoforms. Enhanced oxidative stress and nuclear factor (erythroid 2-related factor) 2 (Nrf2) transcription factor activation have been hypothesised to mediate up-regulation of CYP2A5 expression in the murine liver. The unique and complex regulation of CYP2A5, however, is far from being thoroughly elucidated. Sepsis and high doses of bacterial lipopolysaccharide (LPS) elicit oxidative stress in the liver, but depression, not induction, of CYP2A5 has been observed in studies of mice treated with LPS. The foregoing facts prompted us to evaluate the response of CYP2A5 liver activity in female DBA-2 mice over a broad range of LPS doses (0, 0.025, 0.05, 0.1, 0.2, 0.5, 1, 2, 5, 10, and 20 mg/kg). Cytokine levels (interleukin [IL]-2, IL-4, IL-6, IL-10, IL-17A, interferon gamma, tumour necrosis factor alpha) and nitric oxide (NO) were measured in the blood serum. Activities of CYP1A (EROD) and CYP2B (BROD) in the liver were also determined for comparative purposes. LPS depressed CYP2A5 at low doses (0.025–2.0 mg/kg) but not at doses (>2 mg/kg) that increased pro-inflammatory cytokines and NO serum levels, and depressed CYP1A and CYP2B activities. Blockade of pro-inflammatory cytokines and the overproduction of NO induced by co-treatment with pentoxifylline and LPS and iNOS inhibition with aminoguanidine both extended down-regulation of CYP2A5 to the high dose range while not affecting LPS-induced depression of CYP1A and CYP2B. Overall, the results suggested that NO plays a role in the reversal of the low-dose LPS-induced depression of CYP2A5 observed when mice were challenged with higher doses of LPS.

## Introduction

Cytochrome P450 2A5 (CYP2A5) is a murine enzyme expressed in the liver and olfactory epithelium of the nasal cavity and to a lesser degree in other extra-hepatic tissues such as intestines, oesophagus, lung, and kidneys [1]. It is an orthologue of rat CYP2A3 and human CYP2A6/13 [1,2]. In mice, oxidation of coumarin to 7-hydroxycoumarin is catalysed predominantly by CYP2A5, and thus the coumarin 7-hydroxylase (COH) reaction is considered a selective marker of its activity [3]. CYP2A5 also mediates the metabolism and activation of several toxicants including nicotine, cotinine, aflatoxin B1, coumarin, tobacco-specific carcinogen nicotine-derived nitrosamine ketone, N-nitrosodiethylamine, acetaminophen, and other xenobiotic compounds [2,4]. Where endogenous substrates are concerned, CYP2A5 functions as a bilirubin (BR) oxidase enzyme and converts BR into biliverdin [5,6]. BR is cleared predominantly through biliary excretion of bilirubin glucuronide, the formation of which depends on a conjugation reaction catalysed by uridine diphospho-glucuronosyl transferase 1A1 [7]. Nonetheless, many researchers believe that oxidation mediated by CYP2A5 in mice (or CYP2A6 in humans) contributes to the strict regulation of BR levels in liver tissue to achieve adequate balance between BR antioxidant (low concentrations) and cytotoxic effects (higher levels) [5,6,8,9].

Chemical and pathological modulation of CYP2A5 expression and activity has unique characteristics. CYP2A5 is induced by a variety of structurally unrelated chemicals including heavy metals (Cd, Co, Ce, In, Pb, and methylmercury), hepatotoxic agents (e.g. pyrazole, carbon tetrachloride, chloroform), porphyrinogenic substances (griseofulvin, thioacetamide, and aminotriazole), cell cyclic adenosine monophosphate disruptors (e.g. glucagon, isoproterenol, forskolin), cocaine, and nuclear receptor ligands (e.g. phenobarbital), among other compounds [2,4,10].

Another apparent singularity of CYP2A5 modulation is that its activity, expression, or both are up-regulated in a number of pathophysiological conditions under which most other CYPs are down-regulated. The expression or activity of liver CYPs is generally depressed during aseptic inflammatory processes and infectious diseases including viral, bacterial, and parasitic infections, chronic inflammatory conditions, and challenges with stimuli such as lipopolysaccharide (LPS) and pro-inflammatory cytokines [11–13]. Unlike the expression and activity of most CYP enzymes, however, CYP2A5 activity remains unchanged in the chronic phase of murine schistosomiasis [14] and is up-regulated during malaria [15,16], viral hepatitis B [17,18], liver fluke *Fasciola hepatica* infection [19], fasting [20], bacterial hepatitis [21], and liver cancer [22]. In hamsters, *Opisthorchis viverrini* infection enhances the expression of a protein reactive to antibodies against mouse-CYP2A5 in hepatic tissue [23].

Although some light has been shed on CYP2A5 gene regulation, the cellular events that ultimately lead to CYP2A5 induction in the liver remain largely unknown [2,4]. Kirby and co-workers [4,24,25] proposed that overexpression of CYP2A5 in response to some infections and liver toxicants (e.g., pyrazole) is triggered by pathological changes that occur in hepatocellular injury rather than by events elicited by immunostimulation that eventually lead to the down-regulation of most CYPs. As highlighted in recent reviews of research addressing the regulation of CYP2A5/6 [2,4], accumulating evidence suggests that common gene regulatory mechanisms (e.g., via nuclear factor erythroid 2-related factor 2, Nrf2) are shared by CYP2A5/6 and cellular enzymes that respond to oxidative stress. Moreover, metabolism and homeostasis of haeme are hypothesised to be crucial factors in the regulation of liver CYP2A5 expression mediated by Nrf2 activation [2,4,6,8].

Surprisingly, LPS induction of CYP2A5 has not yet been demonstrated. Sepsis and high-dose LPS challenge reportedly elicit oxidative stress response and induce haeme oxygenase 1 via Nrf2 activation, thereby interfering with haeme metabolism [26–28]. Therefore, if oxidative stress does indeed lead to CYP2A5 up-regulation, enzyme induction would be expected to occur under conditions of sepsis and challenges with high doses of LPS. LPS is one of the most studied inflammatory stimuli and causes a generalized depression of total CYP content and decreased expression and activity of many forms of CYP in the livers of rodents. Gilmore and co-workers [24] noted that messenger RNA (mRNA), protein, and activity (COH) levels of CYP2A5 were depressed by intraperitoneal (i.p.) LPS treatment (0.5 mg/kg body weight [bw]), a down-regulation similar to that reported for many CYP forms after challenge with an inflammatory stimulus. The authors also reported that a dose of LPS 10 times greater (5 mg/kg body weight, i.p.) produced time-dependent depression of CYP2A5 protein levels and activity (COH) that became more marked 24 h after dosing [24].

Experiments carried out at our laboratory demonstrated a marked depression of hepatic CYP2A5 activity in mice challenged with 0.5 mg/kg bw LPS, a finding consistent with those reported by Gilmore et al. [24]. Most animals treated with higher doses of LPS (e.g., 5 and 20 mg/kg bw), however, exhibited no clear decline in CYP2A5 activity, instead showing a consistent decrease of CYP1A (ethoxy-resorufin-*O*-demethylase [EROD]) and CYP2B (benzyloxy-resorufin-*O*-debenzylase [BROD]) activities.

These unexpected results prompted us to examine how CYP2A5 (COH) activity changes over a broad range of LPS doses. We tested the hypothesis that CYP2A5 activity in the liver switches from down-regulation to up-regulation as the strength of LPS-elicited immune stimulation increases from very low doses to high doses compatible with septic shock. We also investigated whether blockade of the LPS-triggered rise in pro-inflammatory cytokines and nitric oxide (NO) blood levels would change the point on the LPS dose-response curve at which down-regulation of CYP2A5 activity transitioned to up-regulation. The effect of the LPS doses on the activities of two CYP enzymes that are known to be down-regulated by inflammatory stimuli, CYP1A1/2 (EROD) and CYP2B9/10 (BROD), were determined for comparative purposes.

## Materials and Methods

### Animals

Eight- to 10-week-old female DBA-2 mice bred by the Oswaldo Cruz Foundation were used. All mice were housed on white wood shavings in standard plastic cages with stainless steel cover lids. The animals were maintained under controlled environmental conditions (12 h light/12 h dark cycle; room temperature, 23 ± 2°C; air relative humidity, approximately 70%) with free access to a commercial rodent pellet diet (Nuvital CR1, Nuvital, Curitiba, PR, Brazil) and filtered tap water. The study protocol was evaluated and approved by the Ethics Committee on the Use of Animals of the Oswaldo Cruz Foundation.

### Chemicals

The following chemicals used in the experiments were obtained from Sigma Chemical Co. (St. Louis, MO, USA): pentoxifylline (PTX, P1784), aminoguanidine (AG, A7009), protein standard (P5619), Bradford reagent (B6916), resorufin (R3257), resorufin benzyl ether (B1532), resorufin ethyl ether (E3763), umbelliferone (U7626), coumarin (C4261), nitrate reductase (N7265), reduced nicotinamide adenine dinucleotide phosphate (β-NADPH, N1630), sodium nitrate (S5506), N-(1-naphthyl)ethylenediamine (N9125), sulphanilamide (S9251), nicotinamide adenine dinucleotide phosphate (β-NADP, N0505), glucose-6-phosphate (G7250), glucose-6-phosphate dehydrogenase (G6378) and *Escherichia coli* LPS (type 0127:B8, L3129). All other chemicals used were of high analytical grade.

### Treatment

Mice received i.p. injections of LPS or phosphate-buffered saline (PBS) only and were killed via cervical dislocation 6, 12, or 24 h after treatment. Blood was taken from the retro-orbital sinus immediately before cervical dislocation. Mice co-treated with PTX and LPS received two injections of PTX (100 mg/kg bw i.p.), one 30 min before and another 30 min after challenge with LPS or vehicle (PBS). AG (50 or 100 mg/kg bw i.p.) was administered immediately after treatment with LPS or PBS. PTX and AG were freshly prepared using sterile PBS solution. Animals were always treated between noon and 2 p.m.

### Preparation of liver microsomal fraction (LMF)

After euthanasia, the mouse livers were quickly removed, freed from fat and extra tissue, weighed, and frozen in liquid nitrogen until further use. The LMF was prepared as described by De-Oliveira et al. [29] with the substitution of Tris (100 mM) KCl (150 mM) buffer (pH 7.4) for sucrose solution. LMF was aliquoted into cryotubes and stored in liquid nitrogen until further use. The protein concentration of LMF was determined using the method of Bradford [30] adapted to a multi-well plate spectrophotometer reader (Spectramax Plus, Molecular Devices, USA).

### Determination of liver monooxygenase activities

COH activity was assayed using the method of van Iersel et al. [31] with a few modifications: 50 mM Tris buffer, pH 7.4, 10 μM coumarin and 0.8 mg/mL protein were added to the microtubes (final volume of 0.5 mL). After a 3-min pre-incubation period, the reaction was initiated by adding an NADPH regenerating system (0.5 mM β-NADP, 10 mM glucose 6-phosphate, 0.5 U/mL glucose 6-phosphate dehydrogenase and 10 mM magnesium chloride) for 10 min at 37°C with shaking. The reaction was stopped by adding 2 N HCl to the microtubes. Twenty minutes after the addition of chloroform, 500 μL of the reaction product, umbelliferone, was transferred to tubes containing 750 μL of a 1.6 M glycine-NaOH solution, pH 10.4, and left to stand for 10 min. The upper phase was then transferred into quartz cuvettes, and umbelliferone levels were measured using a spectrofluorometer (Shimadzu RF5301 PC). The equipment parameters were set as follows: excitation at 355 nm, emission at 460 nm, and band slit width at 3 nm.

BROD and EROD were assayed in 96-well microplates using the method of Kennedy and Jones [32] with modification. The reaction volume in the wells was 270 μL, in which the final concentrations were 5 μM substrate, 0.25 mM β-NADP, 5 mM glucose 6-phosphate, 0.5 U/mL glucose 6-phosphate dehydrogenase, and 2.5 mM magnesium chloride. A constant amount of microsomal protein (0.025 mg) was added to each well. After a 10-min reaction at 37°C in a shaker water bath, acetonitrile was added to a final volume of 360 μL to stop the reaction. The reaction product (resorufin) was measured using a fluorescence plate reader (Spectramax GeminiXS, Molecular Devices, USA) with excitation and emission wavelengths set at 530 nm and 590 nm, respectively.

### Measurement of NO levels in blood serum

NO production was estimated by measuring the total nitrite in serum samples with the Griess method, as described elsewhere [33]. Briefly, 40 μL of each serum sample was incubated overnight at 37°C in a 96-well plate with an equal volume of a cocktail containing 0.5 M KH_2_PO_4_, pH 7.5, milliQ water and nitrate reductase and NADPH to achieve concentrations of 0.4 U/mL and 1 mg/mL, respectively, per well. Subsequently, 80 μL of the Griess reagent (a 1:1 mixture of 0.2% N-[1-naphthyl]ethylenediamine in milliQ water, 2% sulphanilamide in 5% phosphoric acid, 5% phosphoric acid, and milliQ water) were added. The absorbance was measured using a multi-well plate spectrophotometer reader (Spectramax Plus, Molecular Devices, USA) at 540 nm, and the results were expressed as concentration (μM) of nitrite.

### Measurement of cytokine levels in blood serum

Serum levels of tumour necrosis factor alpha (TNF-α), interferon gamma (IFN-γ), and interleukin (IL)-2, IL-4, IL-6, IL-10, and IL-17A were determined using the BD Cytometric Bead Array Mouse Th1/Th2 Cytokine Kit (BD Biosciences, San Jose, CA, USA) according to the manufacturer’s instructions. Briefly, a 25-μL plasma sample was incubated for 2 h at room temperature with 25 μL cytokine capture beads and 25 μL PE detection reagent. After incubation, the samples were washed once with the washing buffer via centrifugation (200 × *g*, 5 min). The supernatants were discarded, and the pelleted beads were resuspended in 300 μL of the washing buffer for analysis on a FACSCalibur flow cytometer (Becton Dickinson, San Jose, CA, USA). The plasmatic concentration of each cytokine (pg/mL) was determined based on the standard curves for the recombinant cytokines provided in the kit.

### Statistical analysis

Data were analysed with one-way analysis of variance followed either by the Dunnett’s or Bonferroni’s post hoc test or by the Kruskal-Wallis test followed by the Mann-Whitney test whenever they did not fit a normal distribution. Statistical evaluation was performed using GraphPad Prism version 5.01 for Windows (GraphPad Software, San Diego, California USA), and differences were considered statistically significant at a value of P < 0.05.

## Results

### Modulation of CYP2A5 activity by LPS: dose-response relationship

**Fig 1:**
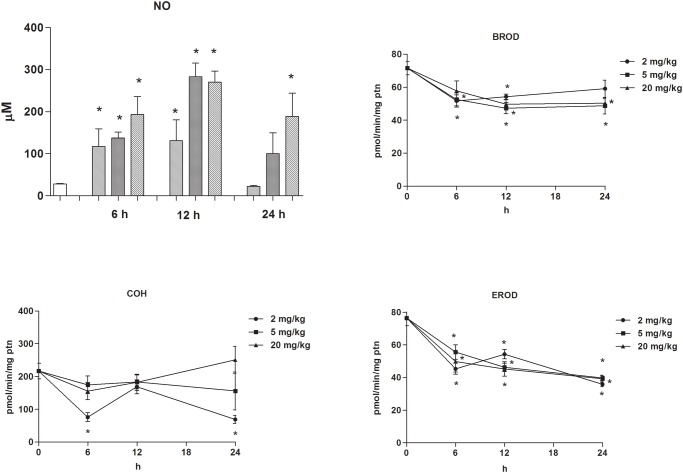
Changes in nitric oxide (NO) concentration in the blood serum and cytochrome P450 (CYP) 2A5, CYP1A, and CYP2B activities in the liver with time elapsed after lipopolysaccharide (LPS) injection. Nitrite concentration (μM), coumarin 7-hydroxylase (COH, CYP2A5), benzyloxy-resorufin-*O*-debenzylase (BROD, CYP2B), and ethoxy-resorufin-*O*-deethylase (EROD, CYP1A) activities (pmol/[min·mg protein]) in the hepatic microsomal fraction of liver samples from female DBA-2 mice injected intraperitoneally (i.p.) with phosphate-buffered saline (control) or LPS (2, 5 or 20 mg/kg) were determined 6, 12 or 24 h after treatment. N = 7 for all groups, except for the control (0 h; N = 20) and LPS (5 mg/kg) groups at 6 h (N = 15). Data represent means ± standard error of the mean (SEM). An asterisk (*) indicates that the mean value differs (analysis of variance [ANOVA] and Dunnett’s multiple comparison test, P < 0.05) from that of the control group.

As shown in Fig. 1, COH activity (indicating a reaction catalysed by CYP2A5) was decreased at the lowest (2 mg/kg bw, 6 and 24 h post-treatment) but not the highest (5 and 20 mg/kg bw) doses of LPS. LPS, on the other side, consistently depressed EROD (a marker for CYP1A/2) and BROD (CYP2B9/10 marker) activities in the mouse liver 6, 12 and 24 h after treatment (Fig. 1).

**Fig 2:**
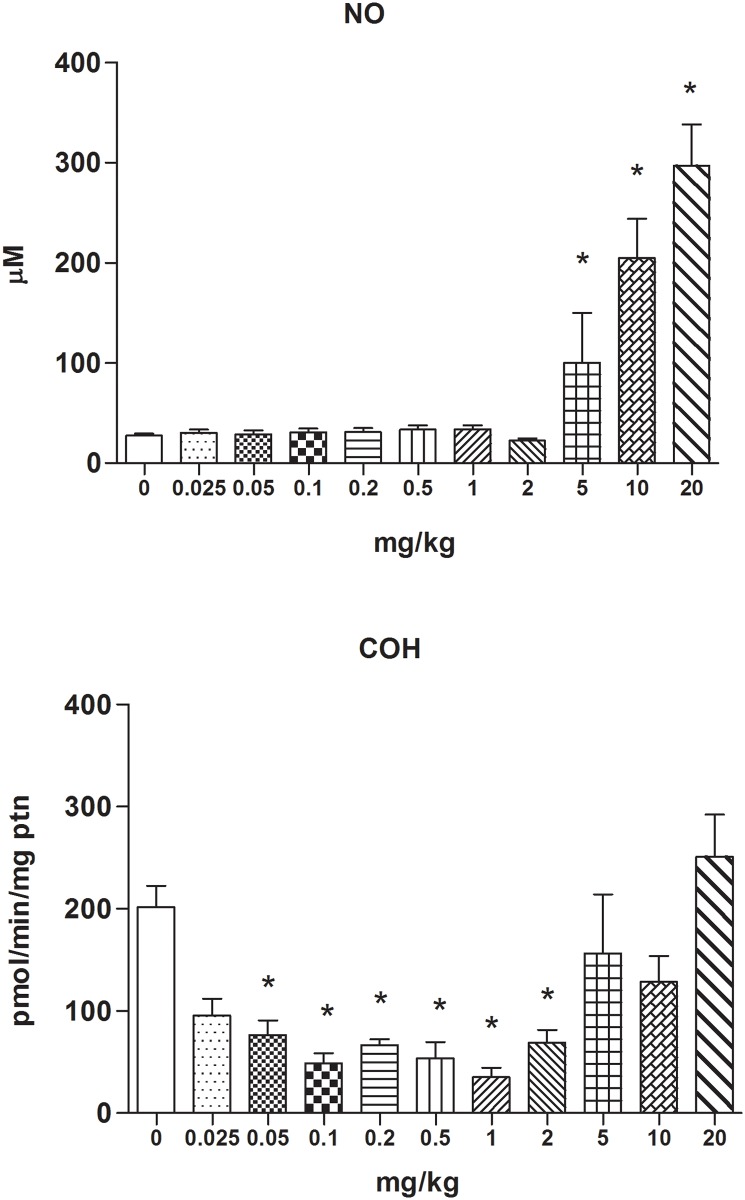
Non-monotonic dose response of CYP2A5 activity in mouse livers after treatment with LPS. NO concentration (μM) and COH activity (pmol/[min·mg protein]) in the hepatic microsomal fraction of liver samples from female DBA-2 mice 24 h after treatment. Mice per LPS dose (mg/kg i.p.) group numbered as follows: 0 (PBS), N = 26; 0.025, N = 3; 0.05, N = 8; 0.1, N = 6; 0.2, N = 6; 0.5, N = 8; 1, N = 6; 2, N = 7; 5, N = 7; 10, N = 10; and 20, N = 7. Data represent means ± SEM. An asterisk (*) indicates that the mean value differs (ANOVA and Dunnett’s multiple comparison test, P < 0.05) from that of the control group.

The foregoing results prompted us to investigate whether down-regulation of CYP2A5 activity by an inflammatory stimulus (e.g., LPS) was in fact a non-monotonic and non-linear dose-response phenomenon. DBA-2 mice were treated with i.p. doses of LPS ranging from 0.025 to 20 mg/kg bw (i.e. 0, 0.025, 0.05, 0.1, 0.2, 0.5, 1, 2, 5, 10 and 20 mg/kg bw), and the NO levels in the blood serum and the activity of CYP2A5 (COH) in the liver were determined 24 h after treatment. The results demonstrated that LPS depressed CYP2A5 activity at low doses (0.025–2 mg/kg bw) but not at high doses (5–20 mg/kg bw). Moreover, LPS diminished CYP2A5 activity at doses below those that elevated serum NO, whereas it did not depress CYP2A5 activity at doses that markedly enhanced NO levels (see Fig. 2).

Data from these two experiments therefore indicated that, in contrast to the monotonic down-regulation of CYP1A and CYP2B as the dose of LPS increases, CYP2A5 activity was depressed by low LPS doses but remained unaltered after (high) doses that elicited marked rises in NO serum levels.

### LPS-mediated down-regulation of CYP2A5 activity after blockade of inflammatory cytokine production with PTX

To verify whether the reversal of low-dose LPS-induced CYP2A5 down-regulation hinged on the enhanced production of NO and pro-inflammatory cytokines, we co-administered LPS with PTX. PTX is a methylxanthine derivative and competitive non-selective phosphodiesterase inhibitor with peripheral vasodilator and anti-inflammatory properties. At the tested doses, PTX did not affect the activities of CYP2A5.

**Fig 3:**
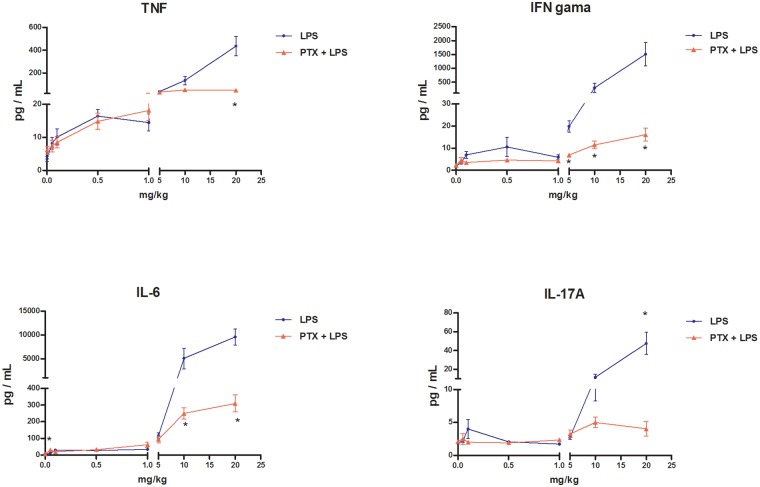
Levels of pro-inflammatory cytokines in the blood serum of mice treated with pentoxifylline (PTX) and LPS. Concentrations of tumour necrosis factor alpha (TNF-α), interferon gamma (IFN- γ), interleukin (IL)-6, and IL-17A were measured in the serum of female DBA-2 mice 24 h after treatment. Animals were treated with LPS (0, 0.05, 0.1, 0.5, 1, 5, 10 or 20 mg/kg i.p., N = 8 per dose group (except the 0 and 20 mg/kg LPS groups, N = 10) alone (●) or PTX plus LPS (2 × 100 mg/kg i.p., 60 min apart, N = 8 per dose group) (▲). Data represent means ± SEM: **a** indicates that the mean differs from the control value in the LPS-treated group; **b** indicates that the mean differs from the control value in the PTX+LPS-treated group; and the asterisk (*) indicates that the mean value in the PTX+LPS-treated group differs from that in the LPS-treated group for the same dose (Kruskal-Wallis and Mann-Whitney tests, P < 0.05).

**Fig 4:**
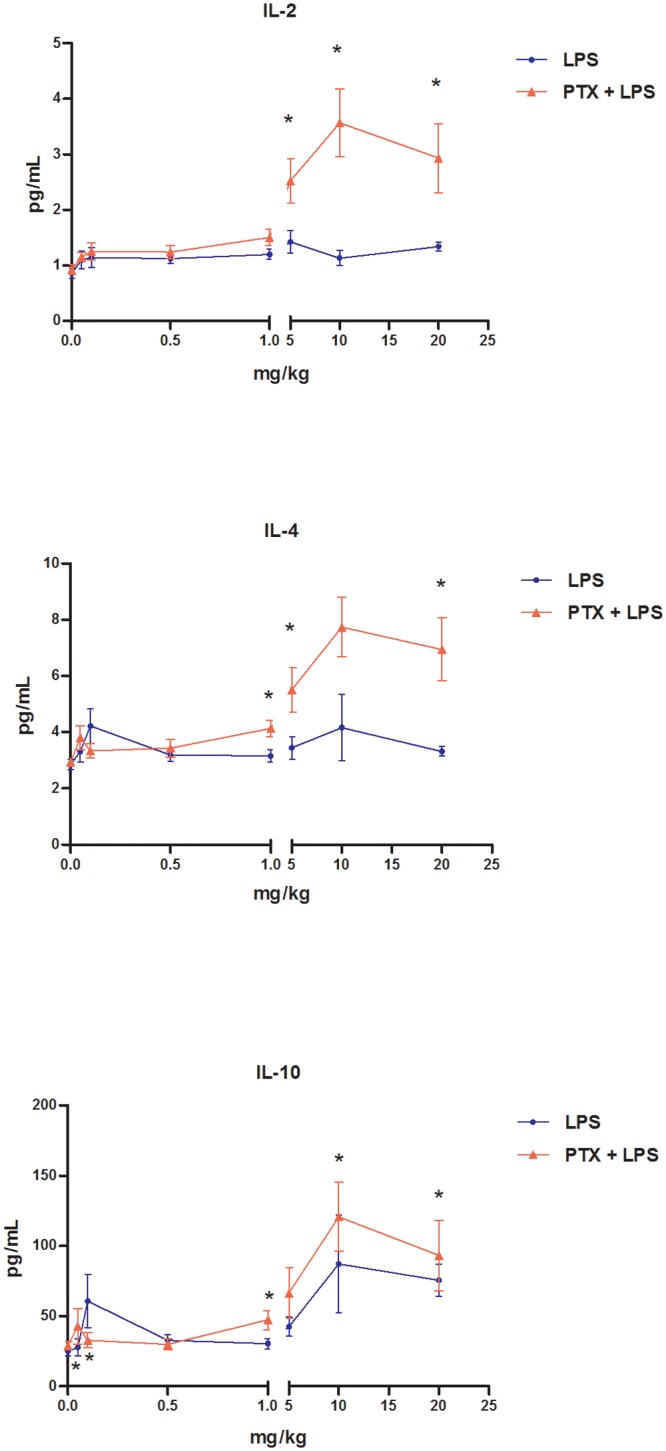
Concentrations of IL-2, IL-4, and IL-10 in the blood serum of mice treated with PTX and LPS. Concentrations of cytokines were measured in the serum of female DBA-2 mice 24 h after treatment. Animals were treated with LPS (0, 0.05, 0.1, 0.5, 1, 5, 10 or 20 mg/kg i.p., N = 8 per dose group [except the 0 and 20 mg/kg LPS groups, N = 10]) alone (●) or with PTX plus LPS (2 × 100 mg/kg i.p., 60 min apart, N = 8 per LPS dose group) (▲). Data are shown as means ± SEM: **a** indicates that the mean differs from control value in the LPS-treated group; **b** indicates that the mean differs from control value in the PTX+LPS-treated group; and the asterisk (*) indicates that the mean value in the PTX+LPS-treated group differs from the mean value in the LPS-treated group for the same dose (Kruskal-Wallis and Mann-Whitney tests, P < 0.05).

**Fig 5:**
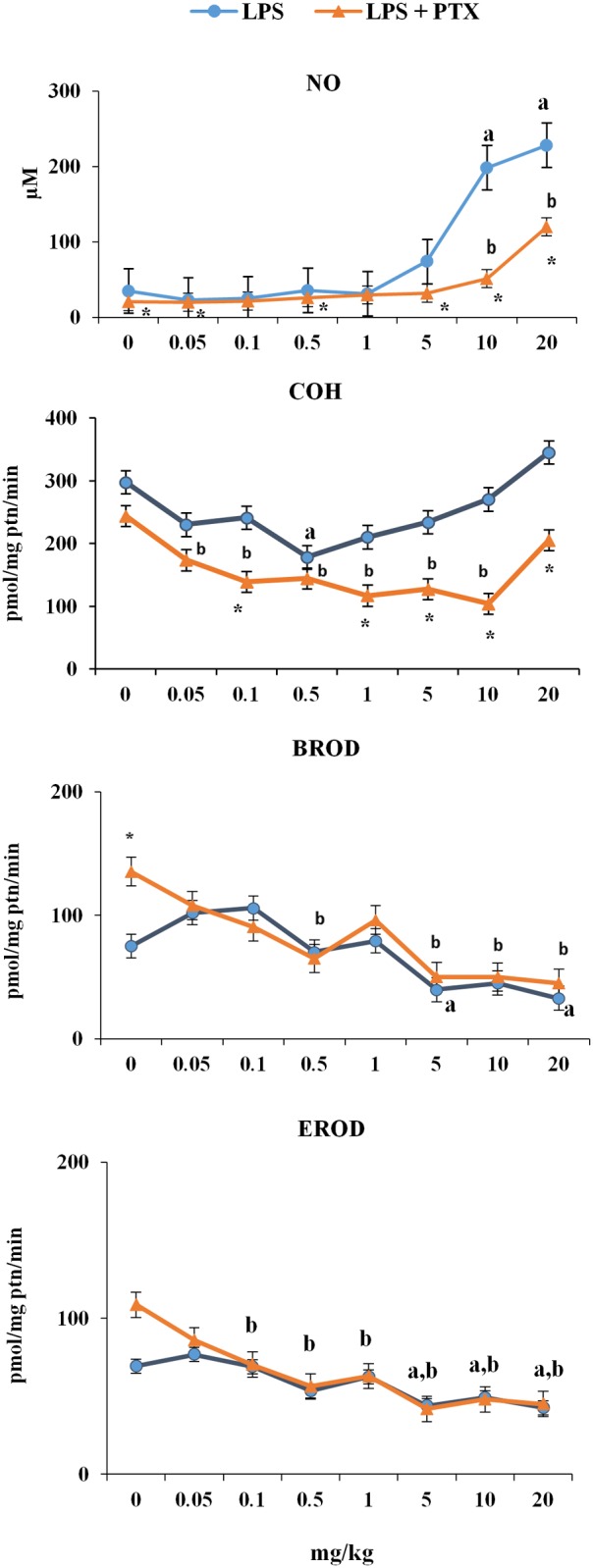
Activities (pmol/[min·mg protein]) of CYP2A5 (COH) and CYP1A and CYP2B (EROD and BROD, respectively) in the liver of mice treated with PTX and LPS. Monooxygenase activities were measured in the liver of female DBA-2 mice 24 h after treatment. Animals were treated with LPS (0, 0.05, 0.1, 0.5, 1, 5, 10 or 20 mg/kg, i.p., N = 10 per dose group) alone (●) or with PTX plus LPS (2 × 100 mg/kg i.p., 60 min apart, N = 8 per LPS dose group) (▲). Data are shown as means ± SEM: **a** indicates that the mean differs from control value in the LPS-treated group; **b** indicates that the mean differs from control value in the PTX+LPS-treated group; and the asterisk (*) indicates that the mean value in the PTX+LPS-treated group differs from the mean value in the LPS-treated group for the same dose (ANOVA and Dunnett’s multiple comparison test followed by the Student’s *t*-test, P < 0.05).

As shown in Fig. 3, co-treatment with PTX (2 × 100 mg/kg bw i.p.) and LPS effectively attenuated the marked rise in serum levels of TNF-α, IFN-γ, IL-6, and IL-17A elicited by LPS at doses of ≥5 mg/kg bw. Conversely, this co-administration increased levels of IL-2, IL-4, and IL-10 compared with those recorded after the administration of LPS alone (Fig. 4). Moreover, co-treatment with PTX and LPS attenuated the sharp rise in NO serum levels induced by LPS at doses of ≥5 mg/kg bw (Fig. 5).

Notwithstanding its anti-inflammatory effect, PTX did not decrease the magnitude of LPS-induced depression of CYP1A1/2 (EROD) and CYP2B9/10 (BROD) activities in the mouse liver (see Fig. 5). PTX-induced blockade of TNF-α, IFN-γ, IL-6, and IL-17A and NO overproduction, however, extended the range of LPS doses that down-regulated the activity of CYP2A5 (COH). LPS-induced depression of CYP2A5 activity also occurred at high doses (5 and 10 mg/kg bw) when endotoxin-elicited NO and pro-inflammatory cytokine overproduction was suppressed by co-treatment with PTX and LPS. At the highest dose tested (20 mg/kg bw) a partial recovery of CYP2A5 activity toward constitutive levels was observed. However, co-administration of PTX and LPS only partially antagonized the marked rise in NO levels triggered by this very high dose of LPS. In other words, regarding CYP2A5 down-regulation, PTX-induced suppression of LPS-elicited inflammatory response (i.e. rise in NO and pro-inflammatory cytokine levels) apparently converted a non-monotonic dose-response curve into a monotonic curve (up to 10 mg/kg bw; see Fig. 5).

### Effect of AG inhibition of NO production on the down-regulation of CYP2A5 by LPS

**Fig 6:**
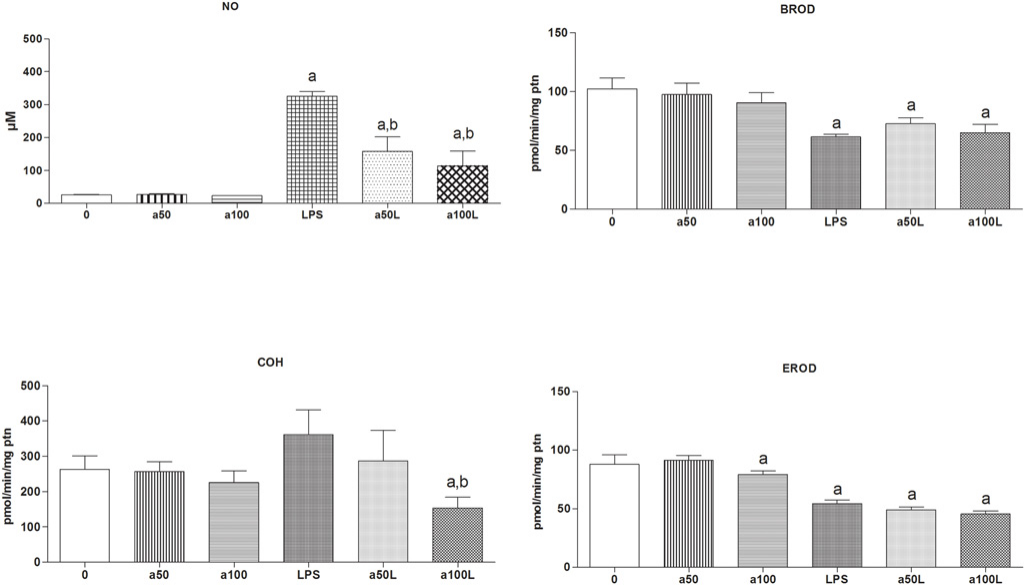
Effect of inducible NO synthase (iNOS) inhibition with aminoguanidine (AG) on LPS-induced changes in CYP2A5 (COH) and CYP1A and CYP2B (EROD and BROD, respectively) activities (pmol/[min·mg protein]) in the mouse liver. NO levels were determined in the blood serum and monooxygenase was measured in liver microsomes from female DBA-2 mice 24 h after treatment. Animals were treated with LPS (0 or 10 mg/kg i.p.) alone or LPS (0 or 10 mg/kg i.p.) plus AG (0, 50 or 100 mg/kg i.p.). The mice per group numbered as follows: 0 (PBS alone), N = 7; LPS alone, N = 9; a50 (AG, 50 mg/kg plus PBS), N = 6; a100 (AG, 100 mg/kg plus PBS), N = 6; a50L (AG, 50 mg/kg plus LPS), N = 6; and a100L (AG, 100 mg/kg plus LPS), N = 6. Data are shown as means ± SEM. **a** and **b** above the bars indicate that the mean values differ (ANOVA and Bonferroni’s multiple comparison test, P < 0.05) from that of the group treated with PBS alone (a), LPS alone (b), or both.

PTX suppresses NO generation by blocking the expression of inducible NO synthase (iNOS or NOS2). To evaluate whether high-dose LPS-elicited NO overproduction plays a role in the shift from low-dose down-regulation to high-dose up-regulation of CYP2A5, we used AG, a selective inhibitor of iNOS activity. As shown in Fig. 6, administration of AG doses as high as 50 and 100 mg/kg bw i.p. did not alter the activities of CYP2A5, CYP1A1/2 or CYP2B9/10 but attenuated the elevation of NO serum levels elicited by a high dose of LPS (10 mg/kg bw i.p.; see Fig. 6). Attenuation of this LPS-induced rise in NO serum levels by AG did not change the magnitude of CYP1A1/2 and CYP2B9 down-regulation. CYP2A5 activity (COH) in mice co-treated with LPS (10 mg/kg bw i.p.) and AG (100 mg/kg bw i.p.), however, was half the activity determined in animals treated with LPS alone (154.1 ± 30 versus 361.5 ± 38 pmol/[min·mg protein]). The foregoing findings are consistent with the notion that down-regulation of liver CYP activities after a challenge with LPS (or other inflammatory stimuli) does not require NO overproduction. Moreover, the effects of post-translation iNOS inhibition with AG (see Fig. 6) and those of pre-translational blockade of iNOS synthesis with PTX (see Fig. 5) are both consistent with the hypothesis that augmented generation of NO contributes to the reversal of CYP2A5 activity depression as LPS dose increases from low to high.

## Discussion

Data from this study showed that LPS-induced down-regulation of CYP2A5 activity in the mouse liver displays non-monotonic dose-response behaviour. Along this line, we demonstrated that low doses of LPS depressed CYP2A5 activity, whereas high doses did not change the activity of this CYP isoform. Moreover, we found that the transition from downward regulation of CYP2A5 activity to upward regulation along the tested dose range coincided with doses of LPS that also enhanced the production of pro-inflammatory cytokines (TNF-α, IFN-γ, IL-6 and IL-17A) and NO. The observation that high doses of LPS (5 and 10 mg/kg bw) also depressed CYP2A5 activity when NO and pro-inflammatory cytokine overproduction was blocked by co-treatment with PTX lent additional support to this interpretation.

Unlike CYP2A5, which was down-regulated by low doses but not by higher doses, CYP1A1/2 (EROD) and CYP2B9/10 (BROD) were depressed by high doses of LPS only. The inhibition of NO and pro-inflammatory cytokine production by LPS and PTX co-administration, however, did not change the magnitude of LPS-induced down-regulation of CYP1A1/2 and CYP2B9/10 activities. These findings are consistent with the notion that the down-regulation of CYP activities does not hinge on the elevation of NO, TNF-α, IFN-γ, IL-6, or IL-17A elicited by inflammatory stimuli (e.g., LPS).

As mentioned, CYP2A5 activity is induced in the murine liver during inflammatory and infectious conditions under which most other CYP activities are down-regulated. The results of this study showed that the same inflammatory stimulus (LPS) either down-regulated or up-regulated CYP2A5 hepatic activity depending on its strength (dose). Additionally, the results demonstrated that discrepancy between CYP2A5 and CYP1A and CYP2B responses exists over a broad range of doses of LPS—i.e., CYP2A5 activity is depressed at doses of LPS at which CYP1A and CYP2B remained unaltered, whereas CYP2A5 activity was unaltered at doses that markedly depressed the activities of the other two CYPs. The point on the dose-response curve at which down-regulation of CYP2A5 turned into up-regulation toward constitutive levels corresponds to the dose that caused a marked rise in blood levels of pro-inflammatory cytokines and NO. Moreover, co-treatment with LPS and PTX shifted both the reversal of the depression of CYP2A5 activity and the sharp increase in pro-inflammatory cytokines and NO blood levels to the right in the LPS dose-response curves.

Notably, PTX did not antagonize LPS-induced down-regulation of CYP1A and CYP2B, and thus blockade of pro-inflammatory cytokines and NO overproduction by co-treatment with LPS and PTX resulted in a considerable overlap between the LPS doses that down-regulated CYP2A5 activity and those that depressed CYP1A and CYP2B activities in the mouse liver. In other words, down-regulation of CYP2A5, CYP1A, and CYP2B activities in the hepatic tissue after LPS challenge seemed not to depend on the endotoxin-elicited rise in cytokines or NO blood levels. Attenuation of LPS-induced NO overproduction by co-treatment with the iNOS blocker AG, however, seemed to decrease CYP2A5 activity compared to that recorded in the absence of AG. This result suggests that the marked increase in NO levels (and not an upstream event in the iNOS induction pathway) may have a role in mediating the transition from down-regulation of CYP2A5 to up-regulation along the LPS dose-response curve. This finding is consistent with the notion that NO-mediated oxidative stress (and Nrf2 activation) [34] plays a role in the up-regulation of CYP2A5 in the murine liver.

The results of previous studies have shown that, although it does not increase NO blood serum levels, malaria infection (*Plasmodium berghei* ANKA) induces CYP2A5 activity in the livers of DBA-2 mice [15,16]. A possible explanation for the absence of an overt increase in NO blood levels in infected animals is the quenching of NO by cell-free haemoglobin released by malaria-induced haemolysis. The markedly enhanced expression of iNOS mRNA in the liver of *P*. *berghei*-infected mice, however, suggests that NO levels are augmented in the vicinity of hepatocytes. Increased lipid peroxidation (thiobarbituric acid-reactive substances) in the hepatic tissue and elevated levels of aspartate aminotransferase and alanine aminotransferase in the blood of infected mice were additional indications that malaria infection enhanced oxidative stress and the degree of liver injury [16].

## Conclusions

This study revealed that liver CYP2A5 activity exhibits a non-monotonic dose-response relationship when mice are challenged with LPS. CYP2A5 is down-regulated by LPS doses that do not alter CYP1A and CYP2B activities and are insufficient to elicit any increase in pro-inflammatory cytokines and NO blood levels. Depression of CYP2A5 activity, however, is attenuated and eventually reversed at higher doses of LPS that down-regulate CYP1A and CYP2B activities and raise blood pro-inflammatory cytokine and NO levels. When overproduction of pro-inflammatory cytokines and NO was blocked by PTX treatment, the activities of CYP1A, CYP2B, and CYP2A5 were all down-regulated by high doses of LPS. This finding suggests that the overproduction of pro-inflammatory cytokines or NO is not required for the down-regulation of CYP1A, CYP2B, and CYP2A5 activities. The overproduction of cytokines and NO elicited by LPS, however, is associated with the up-regulation of CYP2A5 activity toward constitutive or even supra-constitutive levels. Nonetheless, when mice were treated with AG, a selective inhibitor of iNOS, CYP2A5 activity was depressed by a high dose of LPS as well. This finding suggests that NO plays a role in the reversal of low-dose LPS-induced down-regulation of CYP2A5 activity when mice are challenged with higher doses of LPS. High levels of NO in the hepatic tissue are associated with enhanced oxidative stress and Nrf2 activation, and thus these findings seem to support the notion that oxidative stress up-regulates CYP2A5 expression.

A possible explanation for the two opposite and dose-dependent effects of LPS on CYP2A5 activity is that overproduction of NO (due to iNOS induction at high doses of LPS) leads to ER stress that in turn enhances CYP2A5 gene transcription. Enhanced ER-stress has been postulated to mediate the induction of CYP2A5 by pyrazole and inflammatory conditions [24,25,35,36]. In a similar manner, NO has opposite concentration-dependent effects on cell apoptosis, i.e., low NO concentrations protect from apoptosis whereas excessive NO leads to ER-stress and apoptosis. It is generally though that the induction of CCAAT-enhancer-binding protein homologous protein (CHOP) transcription factor by ER-stress leads to NO-mediated cell apoptosis [37,38]. In principle, the excess of NO may also act directly on CYP2A5 protein (at a posttranslational level). Post-translational protein modifications such as nitration (e.g., adding a nitro group to one of the two ortho-carbons of the tyrosine residue aromatic ring) and S-nitrosylation (binding an NO group to a protein cysteine residue) caused by NO and reactive nitrogen species may result in effects such as loss or gain in protein function [34,39,40]. Further studies including in vitro experiments (e.g. with hepatic cell lines) are necessary to shed light on the mode by which LPS and excess of NO modulate CYP2A5 activity.

In conclusion, the results of this study suggest that, similar to most CYP isoforms, CYP2A5 is down-regulated by inflammatory stimuli via a poorly understood link between inflammation-signalling pathways and CYP gene transcription regulation. In the case of CYP2A5, however, up-regulation superimposes the inflammation-caused depression of enzyme activity when inflammatory stimuli are accompanied by overproduction of NO and high blood levels of pro-inflammatory cytokines.
